# Facile Synthesis of Cobalt-Doped Porous Composites with Amorphous Carbon/Zn Shell for High-Performance Microwave Absorption

**DOI:** 10.3390/nano10020330

**Published:** 2020-02-14

**Authors:** Qilei Wu, Huihui Jin, Bin Zhang, Siqi Huo, Shuang Yang, Xiaogang Su, Jun Wang

**Affiliations:** 1 School of Materials Science and Engineering, Wuhan University of Technology, Wuhan 430070, China; wuqilei@whut.edu.cn (Q.W.); Jinhuihui@whut.edu.cn (H.J.);; 2 Ministry of Education Key Laboratory of Textile Fiber Products, School of Materials Science and Engineering, Wuhan Textile University, Wuhan 430200, China; 3 Center for Future Materials, University of Southern Queensland, Toowoomba 4350, Australia; Siqi.Huo@usq.edu.au; 4 School of Mechanical and Electronic Engineering, Wuhan University of Technology, Wuhan 430070, China; ysfrp@whut.edu.cn; 5 Institute of Advanced Material Manufacturing Equipment and Technology, Wuhan University of Technology, Wuhan 430070, China

**Keywords:** MOFs-derived carbon composites, porous structure, impedance match, microwave absorption

## Abstract

A facile method for the preparation of microwave absorbers with low density, high microwave absorptivity, and broad band is of paramount importance to the progress in practical application. Herein, commonly-used metal organic frameworks (MOFs) prepared just by mechanical stirring in methanol at room temperature were chosen as sacrificial templates to synthesize porous carbon composites with tunable dielectric and magnetic properties. With the replacement of Co atoms on the surface of zeolitic imidazolate framework-67 (ZIF-67) by Zn atoms, a Co-doped porous carbon composite with a low-dielectric amorphous carbon/Zn shell was constructed after annealing, leading to excellent impedance matching condition. Consequently, the as-obtained composite (Co/C@C-800) shows marvelous microwave absorption properties with an absorption capacity of −43.97 dB and a corresponding effective absorption bandwidth of 4.1 GHz, far exceeding that of the traditional porous carbon and composites directly derived from ZIF-67. The results provide a convenient way to modify MOFs for enhanced microwave absorption materials from the synergy of dielectric and magnetic losses.

## 1. Introduction

To meet the ever-increasing demand for electronic communications, microprocessors, and digital systems, as well as the increasingly more severe electromagnetic pollution, extensive endeavors of scientists and engineers have been devoted to developing high-performance microwave absorbers [1,2]. Microwave absorbing materials are mainly used in the following areas: military stealth; electromagnetic radiation protection of radio and television stations; electromagnetic radiation protection of industrial, scientific, and medical equipment; electromagnetic radiation protection of household appliances; and electromagnetic radiation protection of office and residential areas. Among varieties of conventional absorbers—ferrite, carbonyl iron powder, magnetic metal nanoparticles, carbon materials, and conductive polymer, for instance—carbon composites absorbers are one of the most cost-effective microwave absorbers because of their chemical and thermal stability, low price, light weight, and controllable micro-structure [3,4,5]. In particularly, materials with a porous multi-interface structure have been considered as potential candidates in many fields owing to their multiple interfaces [6,7,8,9,10]. However, carbon materials’ application is limited by the cumbersome multistep operations.

According to the microwave absorbing theory, the microwave absorbing performance is extremely dependent on the dielectric and magnetic loss, as well as the impedance matching of the microwave absorbers [11,12]. Synthesizing composites doped with other materials [13,14], like magnetic metal nanoparticles, by incorporating magnetic elements to precursors, is a recommendable strategy to prepare carbon composites with enhanced microwave absorption performance. Currently, the research of the most commonly used metal organic frameworks (MOFs) in the field of catalysts has provided inspiration for our work. In particular, the carbonized MOFs can directly obtain porous carbon materials with regular morphology, which simplifies the preparation process, thereby causing great concern in terms of microwave absorption research.

The three-dimensional crystalline networks and metal ions are the keys to the formation of the metal-doped nanoporous structure, leading to better impedance matching and impressive microwave absorption properties [15,16,17]. In our previous work, composites derived from ZIF-67 composites exhibit good magnetic properties with highly porous microstructure, but show exorbitant dielectric constant because of the excessive graphitization [18]. Here, in order to properly weaken the dielectric constant, a porous carbon composite with uniformly-dispersed Co nanoparticles wrapped by a low-dielectric carbon/Zn shell was successfully designed for high-performance microwave absorbers through a simple liquid method using only zinc nitrate methanol solution to generate a shell on ZIF-67. Benefiting from the unique evaporation of zinc metal under high pyrolysis temperature [19,20,21], as expected, a porous low-dielectric amorphous carbon/Zn shell derived from the chelation of zinc ions and 2-methylimidazole was formed to decrease the permittivity for a better impedance match. The detailed data analysis and plenary microscopic morphology characterization demonstrated that the synergistic effect of the low-dielectric shell, internally uniformly-dispersed magnetic Co nanoparticles, high porosity, and large specific surface endows this particular composite with superior microwave absorption performance.

## 2. Experimental Section

### 2.1. Materials

All chemicals in the experiments in were purchased from Aladdin Industrial Co. (Shanghai, China), including cobalt nitrate hexahydrate (Co(NO_3_)_2_·6H_2_O, ≥99.0%), zinc nitrate hexahydrate (Zn(NO_3_)_2_·6H_2_O, ≥99.0%), 2-methylimidazole (≥98.0%), methanol (CH_3_OH, ≥99.9%), and paraffin.

### 2.2. Preparation

**Figure 1:**
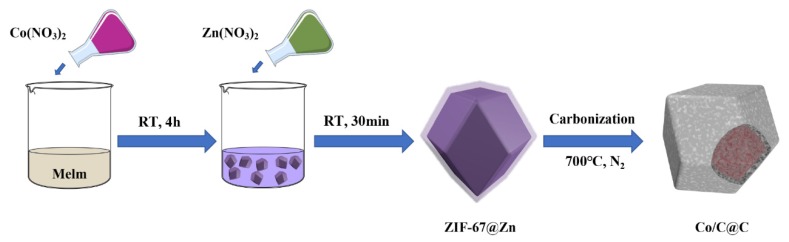
Schematic illustration of Co/C@C fabrication. RT, room temperature.

As shown in Figure 1, 2.5 g of Co(NO_3_)_2_·6H_2_O, 2.5 g of Zn(NO_3_)_2_·6H_2_O, and 5.64 g 2-methylimidazole were dissolved in 135 mL of methanol solution, respectively. Subsequently, 2-methylimidazole methanol solution was slowly and continuously dropped into cobalt nitrate methanol solution, while stirring for 4 h to obtain ZIF-67 methanol solution. Then, the solution of zinc nitrate in methanol was added to the above ZIF-67 methanol solution and stirred vigorously for 30 min. The purple product was collected by centrifugal washing with methanol three times and dried in a vacuum oven at 60 °C for 24 h to obtain ZIF-67@Zn precursor. At last, the prepared precursor was placed in a tube furnace and heated with the heating rate of 5 °C min^−1^ to a set temperature for 2 h under a nitrogen atmosphere (40 mL min^−1^), and finally slowly cooled to room temperature naturally within the tube furnace. Carbonization temperature was carried out at 700 °C, 800 °C, and 900 °C, and the corresponding products were labeled as Co/C@C-700, Co/C@C-800, and Co/C@C-900, respectively.

The control group samples were prepared as the above procedure without the addition of Zn(NO_3_)_2_·6H_2_O, and the carbonization temperature was set at 800 °C, marked as Co/C-800.

### 2.3. Electromagnetic Parameter Measurements

Generally speaking, hysteresis loss, domain wall resonance loss, eddy current loss, and natural resonance loss are the main sources of magnetic loss. The hysteresis loss occurs only at a low frequency and domain wall resonance has an obvious effect under a strong applied field [22,23]. Therefore, eddy current loss and natural resonance are the main forms of magnetic loss in GHz. For eddy current loss, it occurs as the *C*_0_ maintains a constant with the increasing frequency, which can be expressed according to the following equation [24]:

(1) $$C_{0}=\mu^{\prime \prime }{\left(\mu^{\prime }\right)}^{-2}f^{-1}=\frac{2}{3}\pi \mu_{0}d^{2}\sigma$$

where *μ*_0_, *μ′*, *μ″*, *σ*, *d*, and *f* are the relative complex permeability, relative complex permeability, conductivity, sample thickness, and frequency electromagnetic wave, respectively.

The reflection loss (*RL*) is usually adopted to evaluate the electromagnetic absorption capacity. On the basis of transmission line theory, *RL* can be expressed by the following formula [25]:

(2) $$RL=20\log_{10}\left|\frac{Z_{in}-Z_{0}}{Z_{in}+Z_{0}}\right|$$

(3) $$Z_{in}=\sqrt{\frac{\mu }{\varepsilon }}\tanh\left(j\frac{2\pi }{\lambda }d\sqrt{\mu \varepsilon }\right)$$

where *Z*_0_ is the free space impedance; *Z_in_* is the input impedance; *f* is the frequency; *d* is the thickness of absorber; *c* is the light speed; and *ε* and *μ* are the relative complex permittivity and permeability, respectively.

The attenuation factor (*α*) and impedance match are the two most important factors determining the absorbing properties. The attenuation factor (*α*) can be calculated by the following equation [26]:

(4) α=πfc{2(μ″ε″−μ′ε′+((μ″2+μ′2)(ε″2+ε′2))12)}12

Meanwhile, the impedance matching condition can be evaluated by the impedance matching ratio $\left|Z_{in}/Z_{0}\right|$, based on the former calculated input impedance *Z*_0_.

### 2.4. Characterizations

The morphology was characterized by scanning electron microscopy (SEM, S-4800, HITACHI, Tokyo, Japan) and transmission electron microscopy (TEM, JEM-2100F, JEOL, Beijing, China). The crystal phrase structure was analyzed by powder X-ray diffraction (XRD, D8 Advance) in the range of 10–80 degrees with the scan rate of 5°/min. The degree of graphitization of carbon was collected by Raman spectroscopy (INVIAINVIA, RENISHAW, Gloucestershire, UK) with a 633 nm laser. The chemical composition was tested by X-ray photoelectron spectroscopy (XPS, ESCALAB 250XiESCALAB). The vibrating sample magnetometry (VSM, JDAW-2000D, YingPu, Wuhan, China) was used to study the magnetic properties. The N_2_ adsorption/desorption isotherm and pore size distribution were measured at 77 K with ASAP 2020M. Materials’ parameters of standard coaxial ring samples mixed with paraffin were recorded by vector network analyzer (VNA, N5247A, Agilent, Shanghai, China).

## 3. Results and Discussion

**Figure 2:**
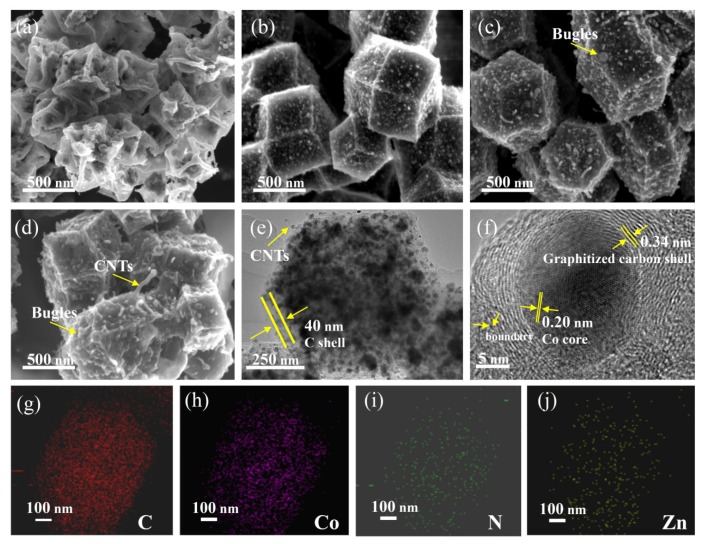
The scanning electron microscopy (SEM) images of (**a**) Co/C-800, (**b**) Co/C@C-700, (**c**) Co/C@C-800, and (**d**) Co/C@C-900. Transmission electron microscopy (TEM) image (**e**), HRTEM image (**f**), and element mapping analysis (**g**–**j**) of Co/C@C-800. CNTs, carbon nanotubes.

The morphology of Co/C-800, Co/C@C-700, Co/C@C-800, and Co/C@C-900 was demonstrated by SEM, as shown in Figure 2a. Co/C-800 prepared without the addition of Zn(NO_3_)_2_·6H_2_O shows a collapsed structure. Significantly, the samples (Co/C@C) prepared with the addition of Zn(NO_3_)_2_·6H_2_O maintain the precursor cubic octahedral structure and are accompanied by a small amount of tubular material anchored on the surface (Figure 2b–d). As the Zn^2+^ is an ideal substitute for Co^2+^ on the surface of ZIF-67, the external surface formed on ZIF-67@Zn can effectively inhibit the collapse of the outer surface carbon skeleton and the destruction of the porous carbon structure during the carbonization. In addition, because of the enrichment of Zn^2+^ on the surface of ZIF-67@Zn, graphitized carbon is not easily formed without sufficient Co catalyst. Therefore, the bubble structure existing on the surface of Co/C@C is formed by low-dielectric carbon/*Zn*. Notably, the difference in temperature has an obvious effect on the morphology of the product. Co/C@C-700 has the largest particle size of about 350 nm, but as the carbonization temperature gradually increases from 700 °C to 800 °C and 900 °C, the particle size of samples gradually decreases to 300 nm and 270 nm, respectively. Besides, the bubble-like bulges on the surface gradually increase with the increasing carbonization temperature, and finally fall apart without obvious shrinkage or collapse.

The TEM test was then conducted to further investigate the microstructure of Co/C@C-800. The outer layer of Co/C@C-800 is a translucent carbon layer of about 40 nm with a small amount of carbon nanotubes, while larger solid nanoparticles are confined to the internal carbon structure (Figure 2e). The additional supplementary enlarged TEM images and EDX images are shown in Figures S1 and S2, respectively. Moreover, the entire composite can be confirmed as a porous structure by N_2_ adsorption–desorption isotherms in Figure S3. The specific surface area of the Co/C@C-800 porous carbon composite is 513.6 m^2^g^−1^ and the average pore size is 5.8 nm. Furthermore, the solid particles with a size of about 15 nm in the composite have characteristic lattice fringes with a spacing of 0.2 nm, and are wrapped by a graphitized carbon layer with a lattice spacing of 0.34 nm (Figure 2f). The element mapping analysis shows that *C*, *N*, and *Zn* are uniformly distributed on the surface of Co/C@C-800 composite, while Co is mainly concentrated inside the composite, indicating that the interior aggregates in the composite are Co nanoparticles (Figure 2g–j). Owing to the limited catalytic activity of metallic Co nanoparticles on amorphous carbon [27,28], few carbon nanotubes are generated and the graphite carbon layers around Co nanoparticles are very thin. The presence of graphitic carbon layer prevents further agglomeration of Co nanoparticles and inhibits the cross-connection between graphitic carbon, so they can be stably and uniformly dispersed in porous carbon [29,30].

**Figure 3:**
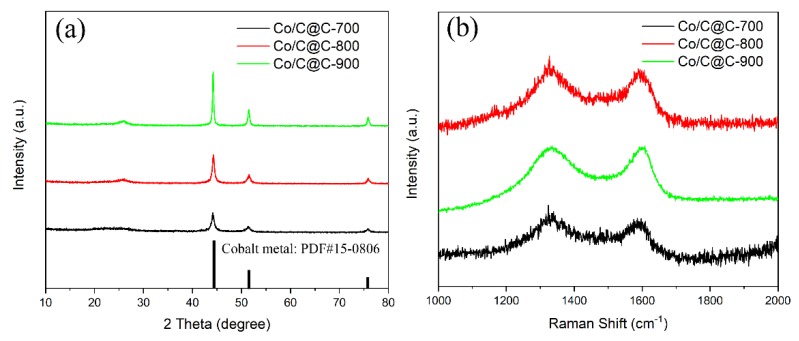
X-ray diffraction (XRD) patterns (**a**) and Raman spectra (**b**) of Co/C@C-700, Co/C@C-800, and Co/C@C-900 composites.

In order to further explore the phase transition process of *C* and Co elements in the carbonization process of ZIF-67@Zn, all the carbonized samples were characterized by XRD. In Figure 3a, three strong diffraction peaks appear at 2*θ* = 44.2°, 52.6°, and 76.0° in all samples, indexing to the face-centered cubic Co metal (111), (200), and crystal faces (220), respectively, based on the PDF card, indicating the conversion of Co^2+^ into Co nanoparticles. The weak diffraction peak at 2*θ* = 25.2° belongs to a highly graphitized face-centered cubic carbon peak, corresponding to the (002) graphite carbon crystal plane. With the increase of carbonization temperature, the graphite carbon peak intensity gradually increases from 700 °C to 900 °C, demonstrating that high temperature condition is beneficial to the graphitization of carbon.

Figure 3b shows the Raman spectra of Co/C@C-700, Co/C@C-800, and Co/C@C-900. All samples have two typical *D* and *G* peaks at 1351 cm^−1^ and 1586 cm^−1^, respectively. The *D* peak mainly represents the disordered structure caused by the amorphous carbon and graphite lattice defects [31]. The *G* peak stands for a regular ordered state of graphitized carbon and the sp^2^ stretching vibration [32]. In general, the degree of graphitization is closely related to the carbonization temperature, and increasing the carbonization temperature can effectively increase the degree of graphitization [33,34,35]. The presence of the *D* peak indicates the existence of a certain degree of amorphous carbon except for graphitized carbon. The peak of amorphous carbon at 2*θ* = 44.0° in the XRD spectrum is hidden by the strong peak of Co at 44.2°. The degree of graphitization can be characterized by the proportion of carbon in the ordered state, generally by the peak intensity ratio of the *D* peak and *G* peak (*I_D_/I_G_*). The *I_D_/I_G_* values of Co/C@C-700, Co/C@C-800, and Co/C@C-900 are 1.21, 1.13, and 1.1, respectively.

**Figure 4:**
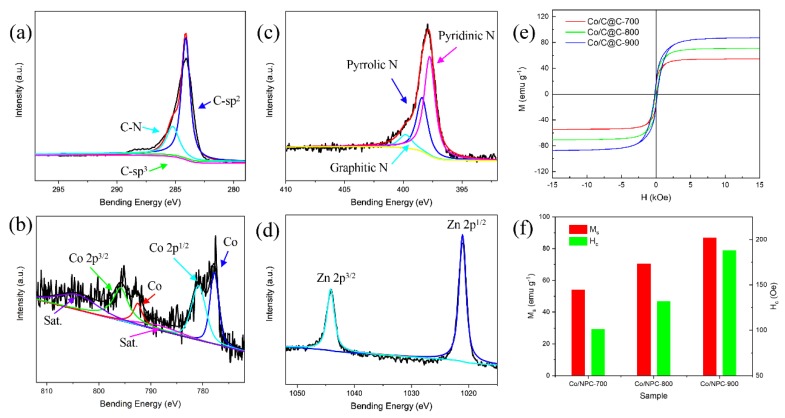
X-ray photoelectron spectroscopy (XPS) spectra of Co/C@C-800 with (**a**) C 1s spectrum, (**b**) Co 2p, (**c**) N 1s, and (**d**) Zn 2p. (**e**) Magnetization hysteresis loops and (**f**) *M_s_* and *H_c_* of Co/C@C-700, Co/C@C-800, and Co/C@C-900.

The surface elemental analysis of the sample Co/C@C-800 was carried out by XPS. The XPS spectrum survey is shown in Figure S4. The peak of C 1s was subjected to peak deconvolution at first (Figure 4a). The three peaks located at 284.6 eV, 284.9 eV, and 286.1 eV correspond to C-sp^2^, C-sp^3^, and C-N bonds, respectively [36]. The peaks of Co 2p include not only Co 2p^1/2^ (796.2 eV), Co 2p^3/2^ (780.9 eV), and two satellite peaks located at 804.1 eV and 788.4 eV, but also the peaks of metallic Co (777.8 eV, 792.5 eV) (Figure 4b) [37]. The energy peaks of N 1s can be divided into three peaks with binding energies of 398.2 eV, 400.1 eV, and 401.1 eV (Figure 4c), corresponding to pyridinic nitrogen, pyrrolic nitrogen, and graphitic nitrogen, respectively [38]. Both pyridinic nitrogen and pyrrolic nitrogen provide a pair of π electrons for the conjugated orbital, and are connected to two carbon atoms in the six-membered ring and five-membered ring, respectively. The graphitic nitrogen directly replaces the carbon atom in the graphene layer. As graphitic nitrogen is highly ordered, nitrogen-substituted atom in the carbocyclic ring can improve the conductivity together with graphitic carbon. Furthermore, graphitic nitrogen, pyridinic nitrogen, and pyrrolic nitrogen as defects can provide polarization sites for electromagnetic waves, thereby further promoting dielectric resonance. As a consequence, the presence of an appropriate *N* element improves the microwave absorption capacity. Besides, Zn 2p can be divided into Zn 2p^1/2^ and Zn 2p^3/2^, as shown in Figure 4d. However, because the Zn^2+^ is reduced and most of them can be volatilized under a high temperature, the small amount of Zn species remaining in the composite cannot be detected by XRD. Interestingly, the Zn atom content (4.06%) on the surface of Co/C@C-800 is almost three times that of the Co atom (1.47%) according to the quantitative analysis of the XPS results, which indicates that the outer surface of Co/C@C-800 has formed a thin layer, leading to the detected Zn atom content being higher than that of the Co atom.

The hysteresis loops and partial enlargements are shown in Figure 4e, and the enlarged images are shown in Figure S5. The coercive force gradually increases with the carbonization temperature and three samples exhibit superparamagnetism at room temperature. As carbon in the components is a non-magnetic substance, the magnetic properties dominantly originate from magnetic substance metal Co. Therefore, the content, crystal form, and particle size of metal Co are three critical factors for the magnetic Co-doped porous carbon composites. Figure 4f shows the magnetic saturation strengths (*M_s_*) of Co/C@C-700, Co/C@C-800, and Co/C@C-900, which are 54.1 emu g^−1^, 70.4 emu g^−1^, and 86.6 emu g^−1^, respectively. The values of coercivity (*H_c_*) are 100.9 Oe, 132.1 Oe, and 188.6 Oe, respectively, higher than that of the bulk cobalt metal. Graphitized carbon on the surface of the main Co nanoparticles can effectively inhibit the agglomeration of the Co nanoparticles, and the size of the Co nanoparticle is about 70 nm, so that there is barely a magnetic domain in a single Co nanoparticle. As the carbonization temperature promotes the growth of the Co nanoparticles, the particle size of the Co nanoparticles becomes larger as the temperature increases, so the magnetic saturation strength increases. Researchers have found that the coercivity *H_c_* of magnetic nanoparticles has a boundary grain size effect [39,40]; when the grain size is less than 70 nm, *H_c_* increases as the magnetic metal nanoparticles size increases. In contrast, when the size of the magnetic metal nanoparticles is larger than 70 nm, *H_c_* decreases as the magnetic metal nanoparticles size increases. At present, the particle size of Co nanoparticles is less than 70 nm, so the particle size and *H_c_* gradually increase with the increase of the carbonization temperature.

**Figure 5:**
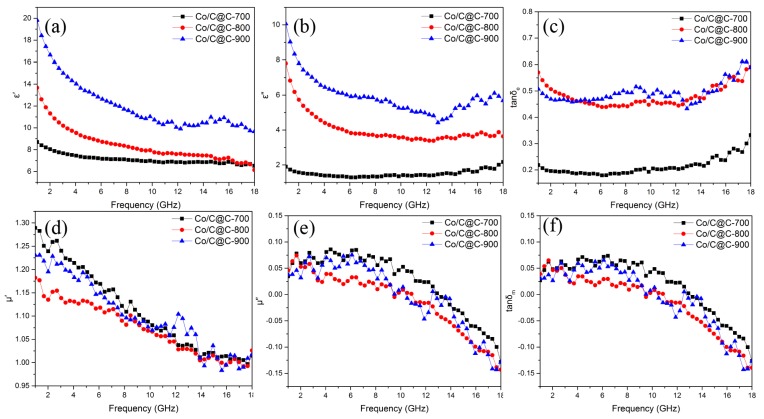
Frequency dependency of (**a**) *ε′*, (**b**) *ε″*, (**c**) *tanδ_e_*, (**d**) *μ′*, (**e**) *μ″*, and (**f**) *tanδ_m_* of as-prepared composites.

In order to measure the electromagnetic parameters, paraffin samples with loading of 15 wt% as-synthesized composites were prepared. The complex permittivity and complex permeability of Co/C@C-700, Co/C@C-800, and Co/C@C-900 composites are shown in Figure 5. The real part of complex permittivity and complex permeability corresponds to the storage capacity of absorbers for the electric and magnetic fields, while the imaginary part represents the loss of the electric and magnetic fields.

Figure 5a,b show the relative complex permittivity of Co/C@C-700, Co/C@C-800, and Co/C@C-900, which presents a tendency to decrease with several fluctuations in the measure frequency range. Co/C@C-700 has the lowest dielectric constant among the three samples, and the real part *ε′* decreases from 8.7 to 7.1, while the imaginary part *ε″* is maintained at approximately 1.6. As the carbonization temperature increases, the dielectric constant increases gradually. *ε′* of Co/C@C-800 gradually decreases from 13.7 to 6.1, as does the imaginary part from 7.8 to 3.6. With the carbonization temperature further increasing, the sample Co/C@C-900 has the highest dielectric constant among the three samples. The corresponding *ε′* reduces from 19.8 to 6.8 and *ε″* from 10.1 to 5.7. According to the theory of free electrons, the complex permittivity is in connection with electrical conductivity, and higher *ε′* corresponds to larger conductivity. As the Co nanoparticles are encapsulated by graphitized carbon and uniformly dispersed in porous carbon, the conductive network cannot be formed effectively. Therefore, the dielectric constant of the material is mainly determined by the graphitized carbon in the sample. The degree of graphitization increases with the carbonization temperature, resulting in the increase of the dielectric constant. In addition, the increasing temperature leads to the formation of more carbon nanotubes on the outer surface, which makes it easier to form an enhanced conductive network by carbon nanotubes between the nanoparticles and increases the electrical conductivity and dielectric constant.

Figure 5c shows the dielectric loss tangent (*tanδ_e_* = *ε″*/*ε′*) of Co/C@C-700, Co/C@C-800, and Co/C@C-900. *tanδ_e_* of Co/C@C-700, Co/C@C-800, and Co/C@C-900 is 0.19–0.20, 0.51–0.55, and 0.47–0.58, respectively, indicating stable dielectric loss in all three samples. Multiple vibrations due to multiple polarizations present in the material are responsible for fluctuations in the curve [41]. The graphitized carbon, amorphous carbon, carbon nanotubes, and Co nanoparticles can form a large number of interfaces with each other. In addition, the defects generated by the nitrogen-doped graphitized carbon and the lattice defect of Co particles also provide the polarization site for applied electromagnetic wave, leading to enhanced dielectric loss. Dielectric loss of the three samples shows a similar increasing trend with increasing carbonization temperature. Because of the lack of shell structure limitation, the probability of cross-linking contact between graphitized carbon and Co nanoparticles in the nanoparticles increases, so the dielectric constant of ZIF-67 derived Co/C-800 is much higher than that of Co/C@C-800, as shown in Figure S6.

The complex magnetic permeability curves of Co/C@C-700, Co/C@C-800, and Co/C@C-900 are shown in Figure 5d–e. It can be seen that the magnetic permeability fluctuates in a small part of the frequency range with a tendency to increase firstly and then decrease as the carbonization temperature increases from 700 °C to 900 °C. The real part *μ′* and the imaginary part *μ″* of the magnetic permeability show a decreasing trend with increasing frequency, and *μ′* and *μ″* fluctuate between 1.14 and 1.26 and −0.14 and 0.06, respectively.

Figure 5f shows the variation of the magnetic loss tangent (*tanδ_m_* = *μ″*/*μ′*). The curve of *tanδ_m_* is similar to the imaginary part of complex permeability, and the vibration decreases between −0.13 and 0.06 as the frequency increases. There is no significant difference in the magnetic loss tangent of the samples, indicating the similar properties of electromagnetic waves loss. The magnetic Co nanoparticles in the sample are all coated with non-magnetic graphitized carbon, which inhibits the interaction between the magnetic crystals of the magnetic Co nanoparticles, and reduces the synergistic loss of electromagnetic waves in the internal magnetic domains. Although the individual magnetic Co nanoparticles size can be improved by increasing the temperature, the formation of graphitized carbon on the Co surface further suppresses the interaction between the neighbouring Co nanoparticles. Thus, samples carbonized at different temperatures have similar magnetic permeability and magnetic loss. In comparison with Co/C-800 in Figure S5, the complex permeability of Co/C@C-800 is similar with Co/C-800, which can be attributed to the low loading of magnetic particles and the same carbonization temperature.

Figure S7 shows the *C*_0_ value of Co/C@C-700, Co/C@C-800, and Co/C@C-900. It is obvious that *C*_0_ is an approximate constant within 6–18 GHz, indicating the eddy current loss in high frequency. In addition to the eddy current loss, there is a tiny peak around 1.5 GHz, corresponding to the natural resonance loss in low frequency.

**Figure 6:**
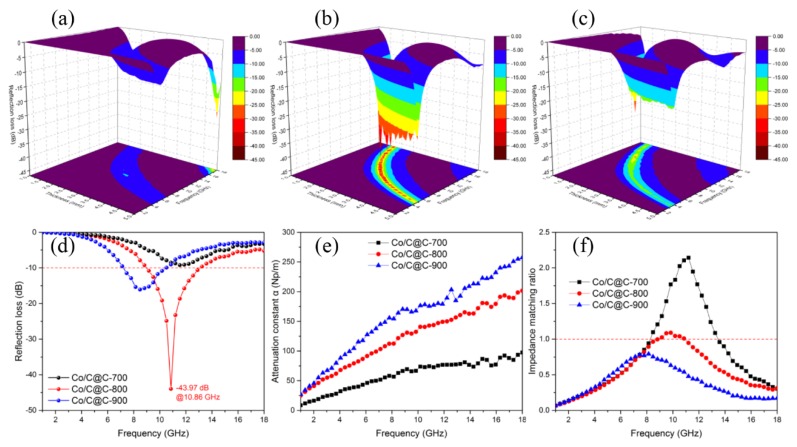
Three-dimensional map reflection loss of (**a**) Co/C@C-700, (**b**) Co/C@C-800, and (**c**) Co/C@C-900. (**d**) Reflection loss, (**e**) attenuation constant *α*, and (**f**) impedance matching ratio $\left|Z_{in}/Z_{0}\right|$ of Co/C@C composites at *d* = 2.5 mm.

Figure 6a–c shows the 3D map RL of Co/C@C-700, Co/C@C-800, and Co/C@C-900 composites. From the above analysis of the significant effects of carbonization temperature on the composition, structure, and electromagnetic parameters, the carbonization temperature is a decisive factor for the microwave absorbing ability. It is obvious that Co/C@C-800 shows the strongest absorbing ability, while Co/C@C-700 exhibits the weakest. The maximum reflection loss of the three samples moves toward a low frequency as the thickness of the sample increases, in accordance with the quarter-wavelength matching model [42]. Although the sample Co/C@C-900 has the largest *ε′* and *ε″*, the absorbing performance is still weaker than that of Co/C@C-800. The excessively higher dielectric constant of Co/C@C-900 than that of Co/C@C-800 accounts for the poor impedance match of Co/C@C-900. The proper carbonization temperature leads to the proper dielectric constant and simultaneously maintains the carbon skeleton structure of the cubic octahedron. The low degree of graphitization of carbon in the shell structure has a lower dielectric constant, which effectively reduces the dielectric constant of the ZIF-67 derived composites and is beneficial to the impedance match. In addition, the presence of the shell structure enables as-prepared composites to maintain a complete cubic octahedral morphology, allowing incident electromagnetic waves to be multiple-reflected within the porous structure. Because of the excessive dielectric constant generated by a lack of low dielectric shell structure, the absorption ability of Co/C-800 composites in Figure S8 is significantly inferior to that of Co/C@C-800 composites.

**Figure 7:**
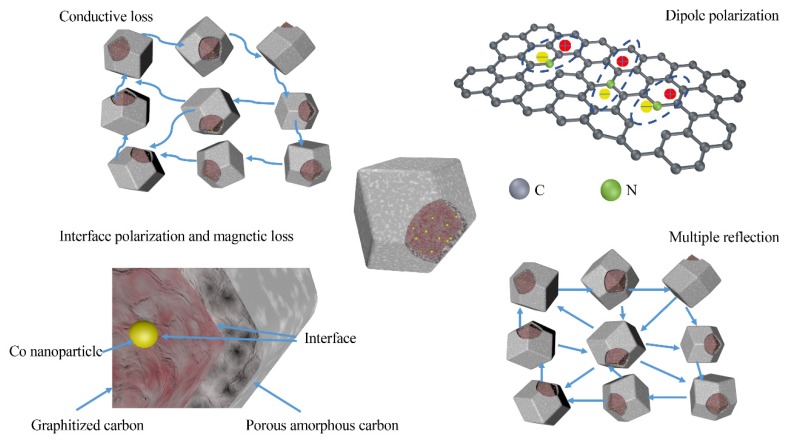
Microwave absorption mechanisms of Co-doped porous carbon composites.

Figure 6e–f show the attenuation constant *α* and impedance matching ratio of the Co/C@C-700, Co/C@C-800, and Co/C@C-900 composites. It is apparent that the *α* values of the three samples become larger as the frequency increases. Co/C@C-900 exhibits the largest attenuation factor, indicating that Co/C@C-900 has the highest electromagnetic wave loss property. In order to explain the phenomenon that the absorbing loss of Co/C@C-800 is stronger than that of Co/C@C-900, the impedance match should be taken into consideration. The impedance matching condition determines whether electromagnetic waves can enter the interior of the material. If electromagnetic waves cannot enter the interior, the attenuation factor has no effect on the absorbing properties. As the input impedance *Z_in_* is equal to the free space impedance *Z*_0_, zero reflection generates with a perfect impedance match. It is distinct that the impedance match of Co/C@C-900 is poor compared with Co/C@C-800 composites. Figure 7 exhibits the microwave absorption mechanisms of Co-doped porous carbon composites. In general, there are three main reasons for the excellent microwave absorption performance of Co/C@C-800 composites: (1) the appropriate carbonization temperature can regulate the graphitized carbon content to obtain an appropriate dielectric constant, leading to an improved impedance match; (2) the shell structure facilitates as-synthesized composites to maintain a porous carbide cubic octahedral structure, which is good for multiple reflection and scattering of incident electromagnetic waves [43,44]; (3) the incorporation of magnetic Co nanoparticles with dielectric carbon improves the impedance match and enhances the microwave energy loss ability, including conductive loss, magnetic loss, dipole polarization, and interfacial polarization loss [45].

## 4. Conclusions

In order to reduce the dielectric constant of ZIF-67-derived composites and improve the impedance matching condition, ZIF-67@Zn was prepared by substituting Zn^2+^ for the Co^2+^ on the ZIF-67 surface, and the low graphitization carbon layer formed on the surface of Co/C@C after carbonization reduces the dielectric constant effectively. The carbonization temperature is the main influencing factor of the dielectric constant of Co/C@C composites. The low dielectric shell structure obtained at a high temperature is responsible for the enhanced microwave absorption performance. The resultant Co-doped porous carbon composites with low-dielectric amorphous carbon/Zn shell (Co/C@C-800) show the best RL of −43.97 dB at 10.86 GHz with an effective absorbing bandwidth of 4.1 GHz. Therefore, this work shines a bright future in lightweight microwave absorption application.

## Supplementary Materials

The following are available online at https://www.mdpi.com/2079-4991/10/2/330/s1, Figure S1: The TEM image and HRTEM image of Co/C@C-800. Figure S2: The element mapping analysis of Co/C@C-800. Figure S3: (a) N_2_ adsorption-desorption isotherms and (b) pore size distribution of Co/C@C-800. Figure S4: XPS spectrum survey of Co/C@C-800. Figure S5: Enlarged magnetization hysteresis loops of Co/C@C-700, Co/C@C-800 and Co/C@C-900. Figure S6: Frequency dependency of (a) *ε′*, (b) *ε″*, (c) *μ′* and (d) *μ″* of Co/C-800 Co/C@C-800. Figure S7: The plot of *C*_0_ as a function of frequency for as-prepared composites. Figure S8: Reflection loss curves of Co/C-800.
