# Supplementary material for: Facile Synthesis of Cobalt-Doped Porous Composites with Amorphous Carbon/Zn Shell for High-Performance Microwave Absorption
Source: Nanomaterials (Basel). 2020 Feb 14;10(2):330. doi: 10.3390/nano10020330 (PMC7075165; doi:10.3390/nano10020330)
Supplement: Supplementary file 1 [file nanomaterials-10-00330-s001.pdf]

Facile Synthesis of Cobalt-doped Porous Carbon Composites with Low-Dielectric Shell for High-Performance Microwave Absorption

Qilei Wu [1], Huihui Jin [1], Bin Zhang [2,*], Siqi Huo [3], Shuang Yang [4], Xiaogang Su [1], and Jun Wang [1,5,*]

1. School of Materials Science and Engineering, Wuhan University of Technology, Wuhan 430070, China; wuqilei@whut.edu.cn (Q.W.); Jinhuihui@whut.edu.cn (H.J.); gang@whut.edu.cn (X.S.)
2. Ministry of Education Key Laboratory of Textile Fiber Products, School of Materials Science and 3 Engineering, Wuhan Textile University, Wuhan 430200, China
3. Center for Future Materials, University of Southern Queensland, Toowoomba 4350, Australia; Siqi.Huo@usq.edu.au
4. School of Mechanical and Electronic Engineering, Wuhan University of Technology, Wuhan 430070, China; ysfrp@whut.edu.cn
5. Institute of Advanced Material Manufacturing Equipment and Technology, Wuhan University of Technology, Wuhan 430070, China

*Correspondence: wgdfrp@whut.edu.cn (J.W.); zhangbin@wtu.edu.cn(B.Z.)

1. Supplementary Figures and Tables

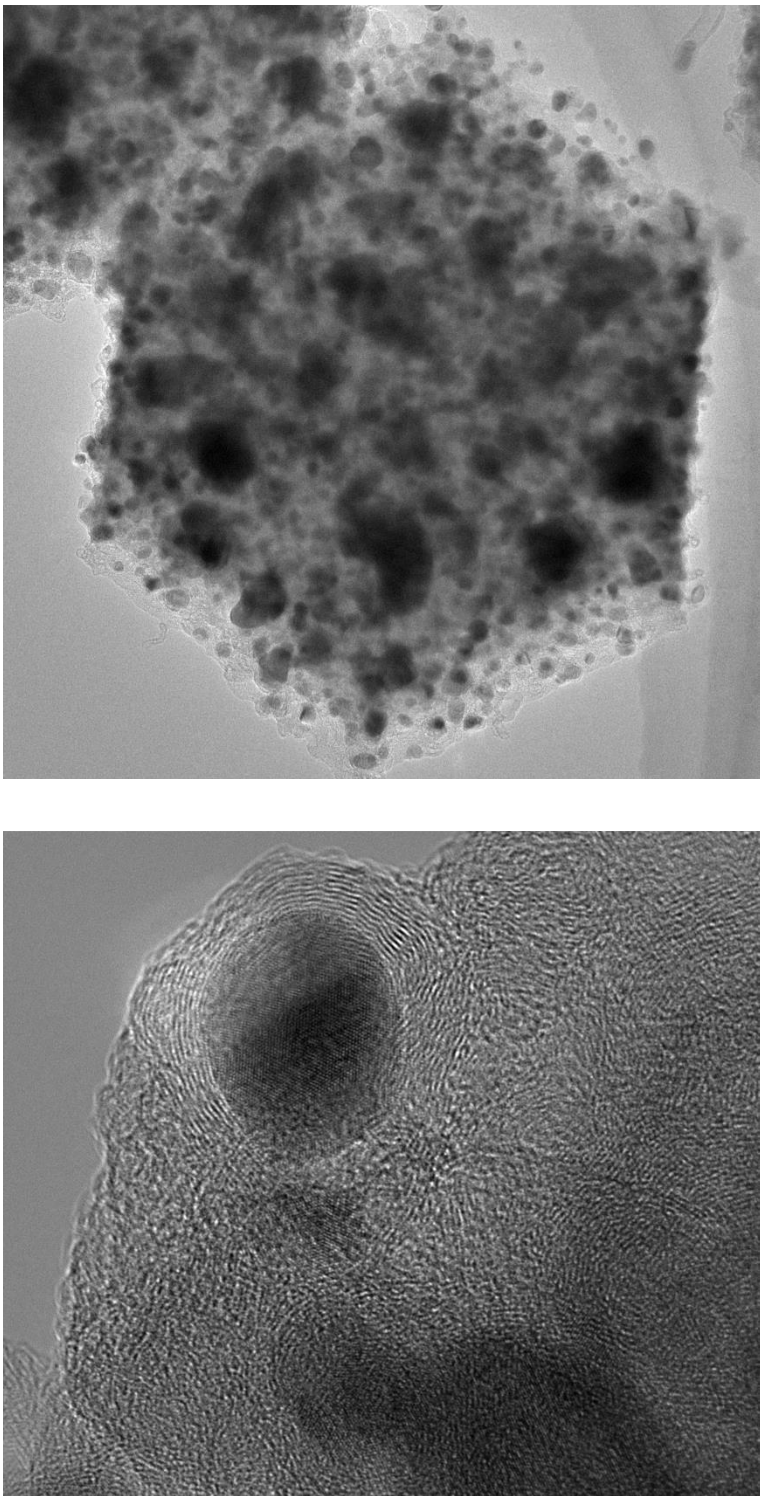

Fig. S1. The TEM image and HRTEM image of Co/C@C-800.

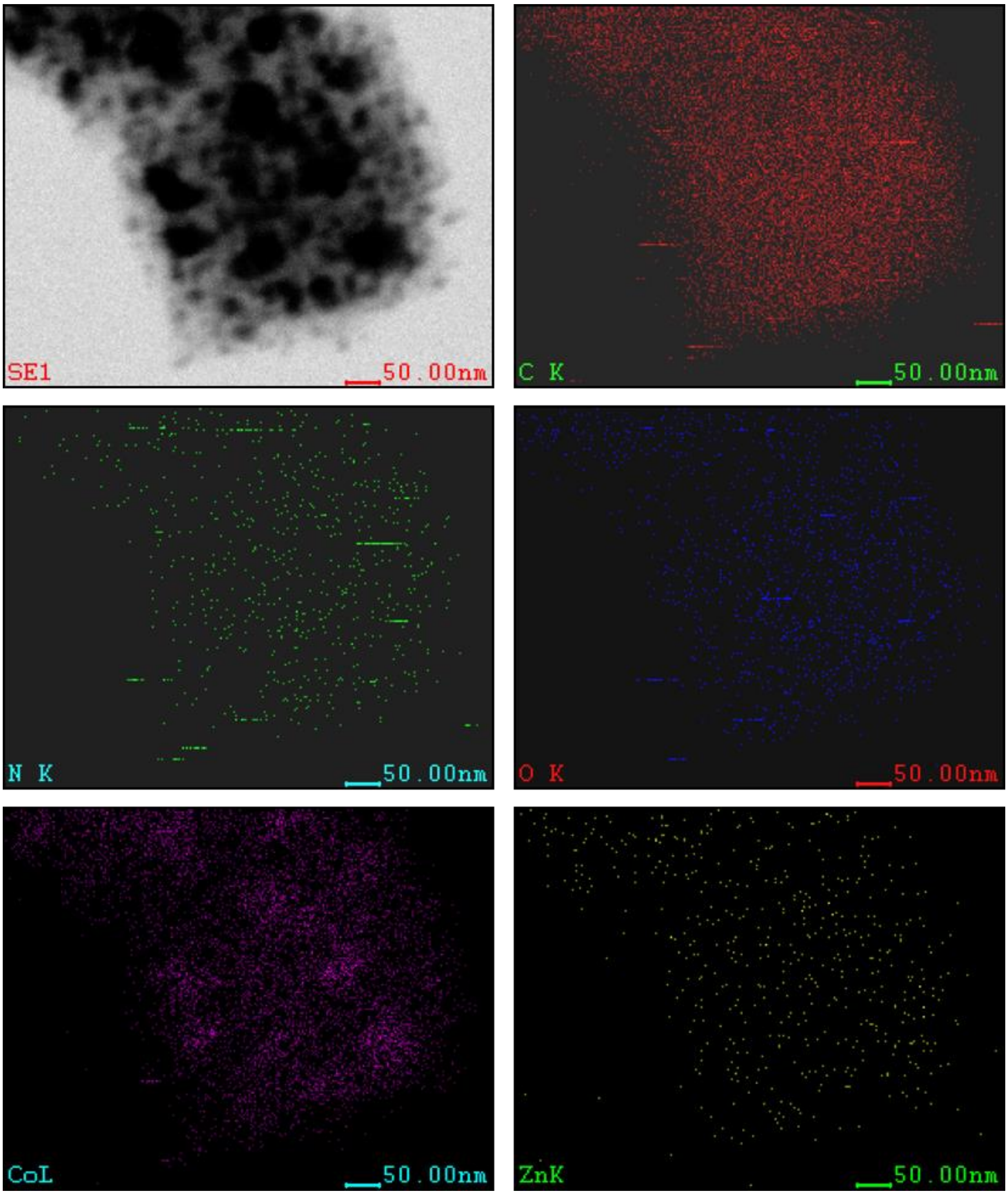

Fig. S2. The element mapping analysis of Co/C@C-800.

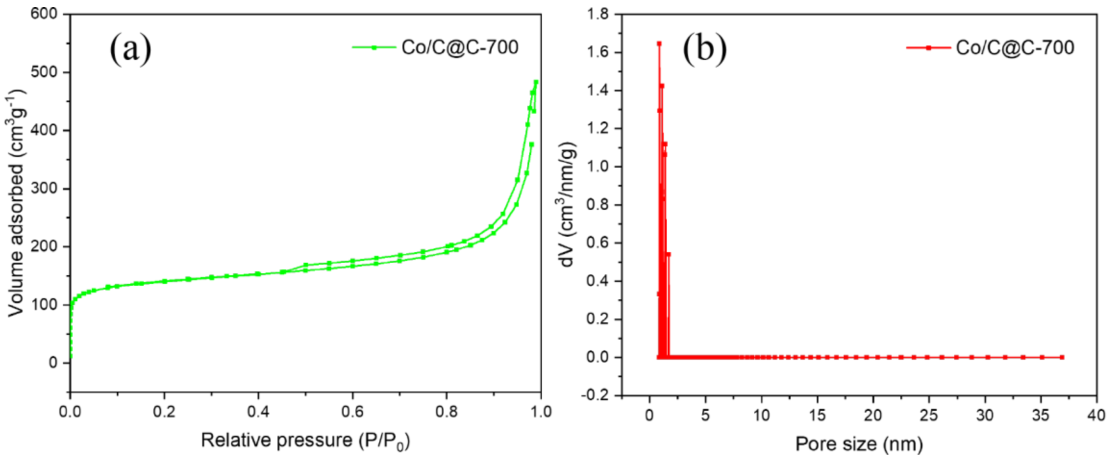

Fig. S3. (a) N_2 adsorption-desorption isotherms and (b) pore size distribution of Co/C@C-800.

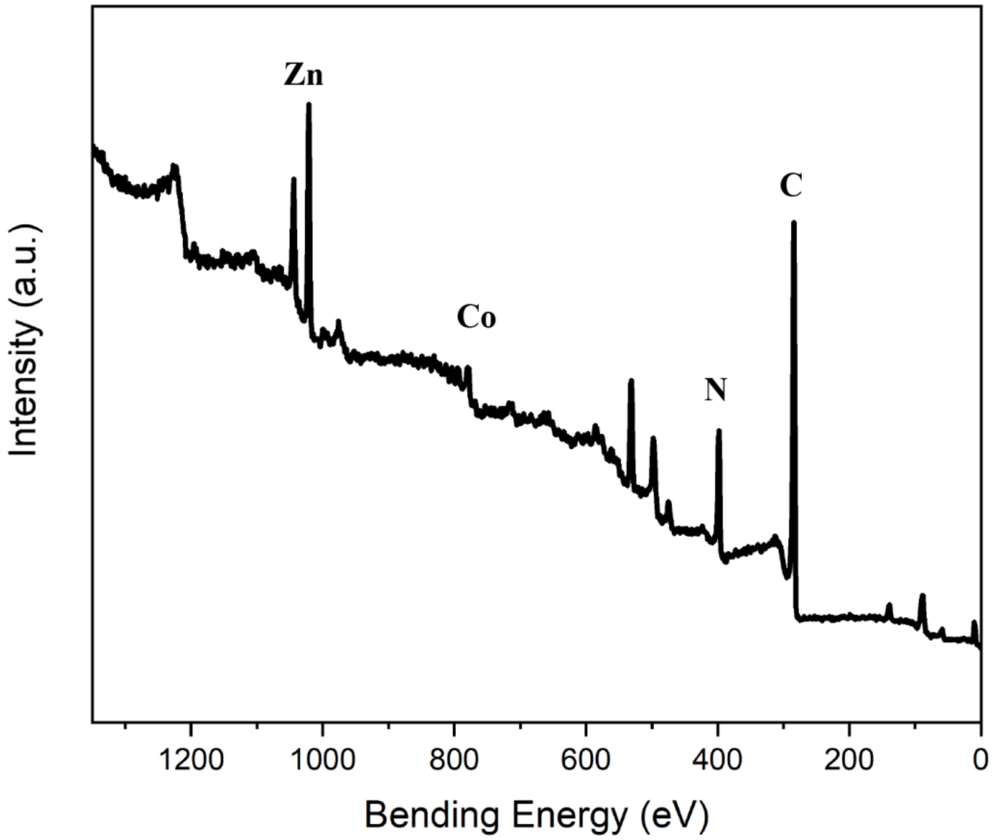

Fig. S4. XPS spectrum survey of Co/C@C-800.

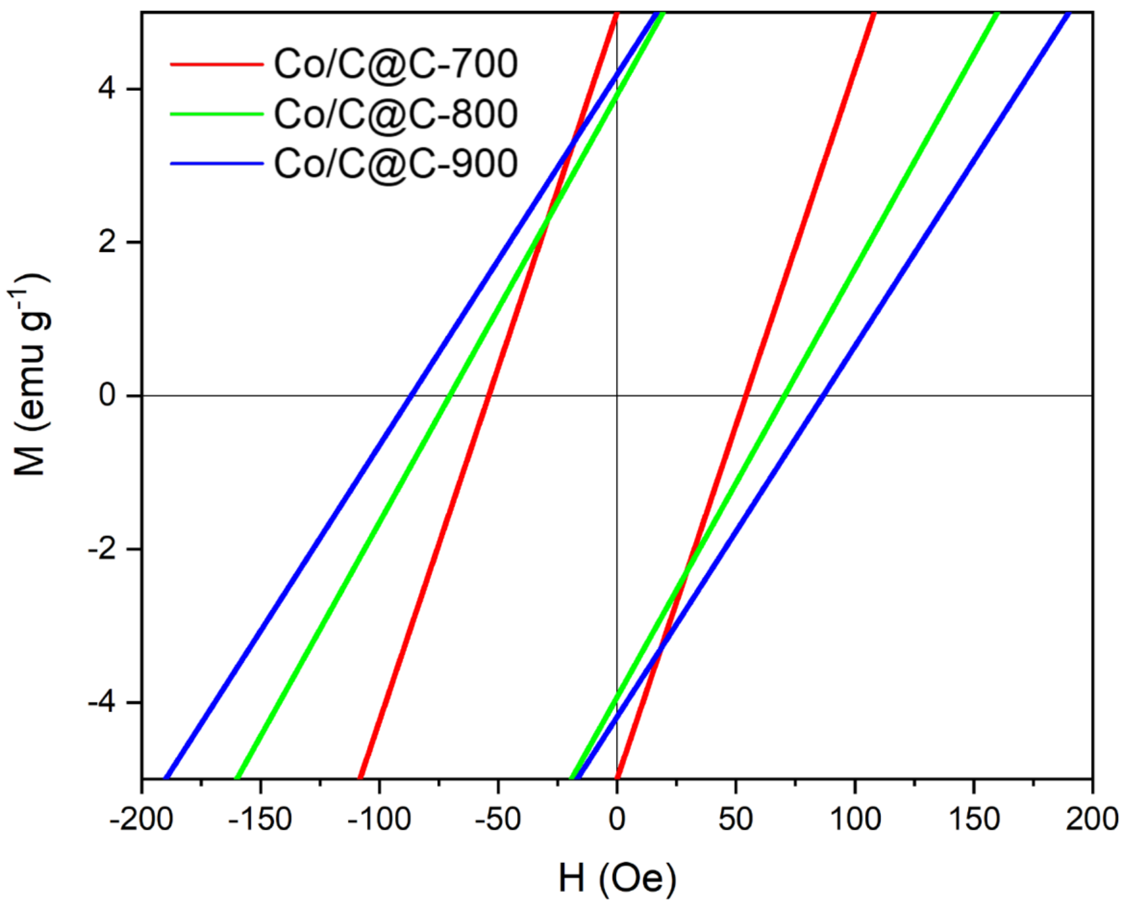

Fig. S5. Enlarged magnetization hysteresis loops of Co/C@C-700, Co/C@C-800 and Co/C@C-900.

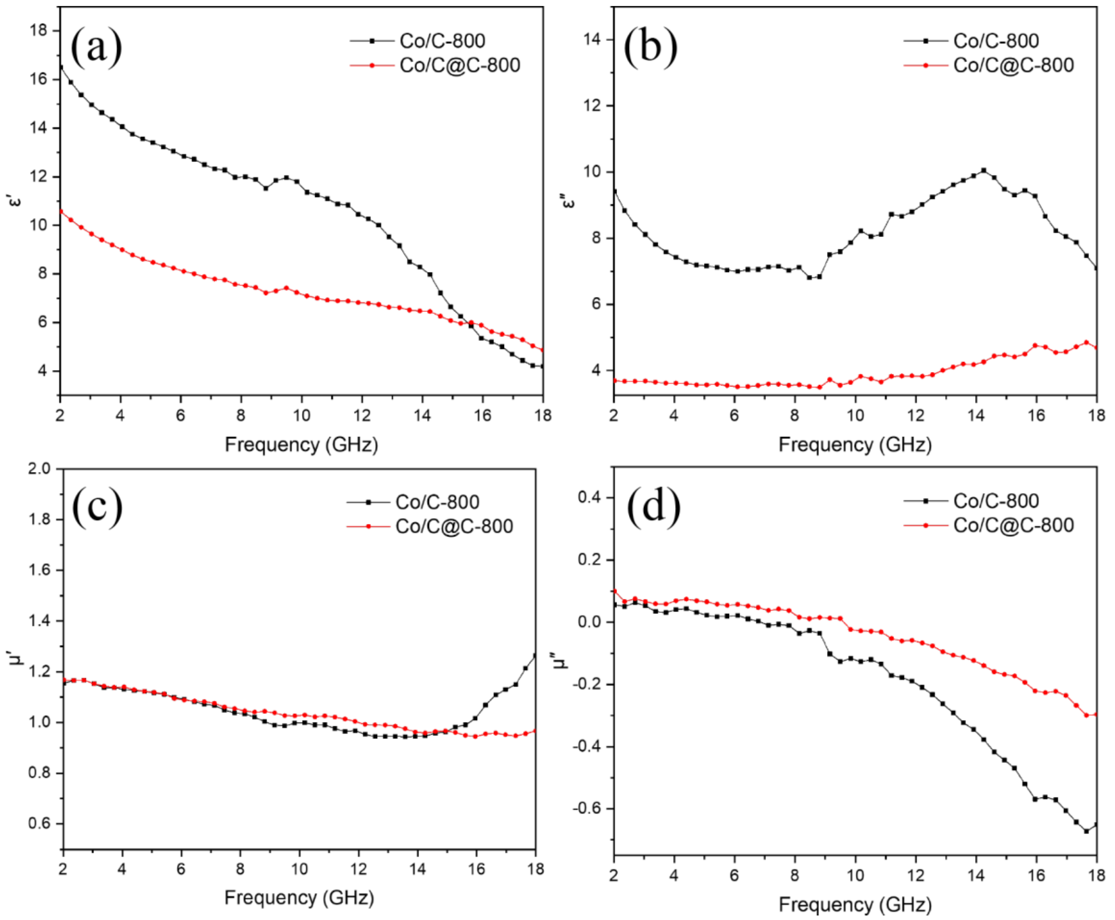

Fig. S6. Frequency dependency of (a) ε', (b) ε'', (c) μ' and (d) μ'' of Co/C-800 Co/C@C-800.

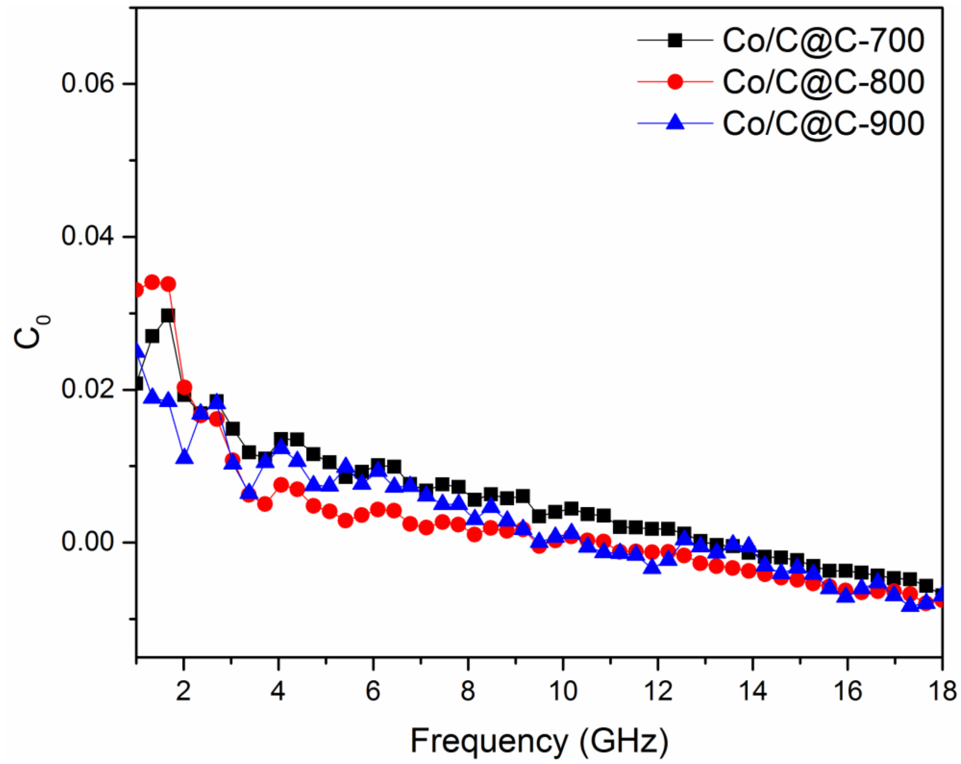

Fig. S7. The plot of C_0 as a function of frequency for as-prepared composites.

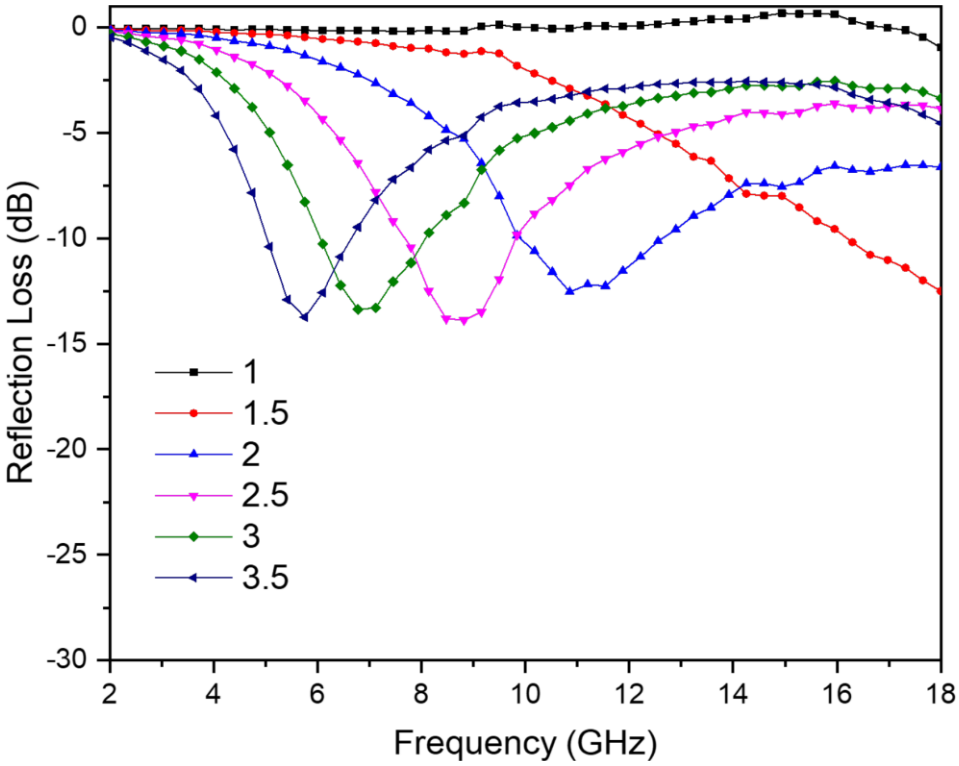

Fig. S8. Reflection loss curves of Co/C-800.
